# Pregnancy Exposure to Polycyclic Aromatic Hydrocarbons (PAHs): Exploratory Comparative Levels in Blood Serum Samples from Different Regions in Antioquia, Colombia

**DOI:** 10.3390/jox16040124

**Published:** 2026-07-02

**Authors:** Jhon Fredy Narváez-Valderrama, Juan José García-Londoño, Juan David González-Calderón, Yileni Argoti-Ospina, Gabriel Jaime Maya, Jorge L. Gallego, Ana Luisa Urrego, Carlos Daniel Ramos-Contreras

**Affiliations:** 1 Grupo de Investigación Ingeniar, Facultad de Ingenierías, Corporación Universitaria Remington, Calle 51 No. 51-27, Medellín 050012, Colombia; yileni.argoti.1439@miremington.edu.co; 2 Grupo de Investigación Materialografía, Transición Energética y Ambiente (MATREA), Facultad de Ingenierías, Corporación Universitaria Remington, Calle 51 No. 51-27, Medellín 050012, Colombia; juan.garcia.3750@miremington.edu.co; 3 Grupo de Investigaciones Biomédicas UniRemington, Corporación Universitaria Remington, Calle 51 No. 51-27, Medellín 050012, Colombia; juan.gonzalez01@uniremington.edu.co (J.D.G.-C.); anaurrego10@gmail.com (A.L.U.); 4 Grupo de Investigaciones y Mediciones Ambientales GEMA, Facultad de Ingenierías, Universidad de Medellín, Carrera 87 No. 30-65, Medellín 050026, Colombia; gjmaya@udemedellin.edu.co; 5 Biodiversity, Biotechnology and Bioengineering Research Group—GRINBIO, Department of Engineering, University of Medellin, Medellín 050026, Colombia; jlgallego@udemedellin.edu.co; 6 Grupo de Investigación en Gestión y Modelación Ambiental—GAIA, Facultad de Ingeniería-Escuela de Microbiologia, Universidad de Antioquia—U.de.A, Calle 70 No 52-21, Medellín 050010, Colombia

**Keywords:** air pollution, bioaccumulation, Biomonitoring, environmental health risk, obstetric perinatal outcomes

## Abstract

Maternal exposure to polycyclic aromatic hydrocarbons (PAHs) during pregnancy has been associated with adverse obstetric and perinatal outcomes, including miscarriage, low birth weight, intrauterine growth restriction, and spontaneous abortion. Exposure occurs through multiple pathways, including dietary intake and inhalation, which ultimately determine the final body burden. PAHs may reach relevant levels in the blood, representing the initial step in their internal distribution to the placenta, umbilical cord, and breast milk, thereby compromising maternal–fetal health. In this exploratory study, maternal blood samples were collected from pregnant women residing in different regions of Antioquia, Colombia. Serum was isolated from whole blood, subsequently extracted using freezing-assisted liquid–liquid extraction, purified by solid-phase extraction, and analyzed by GC–MS. Method performance showed PAH recoveries between 60 and 120%, limits of detection (LOD) ranging from 0.5 to 3.3 ng·mL^−1^, and limits of quantification (LOQ) ranging from 1.4 to 9.9 ng·mL^−1^. Airborne PAH concentrations were measured using a photoelectric aerosol sensor, and higher levels were observed in municipalities intersected by major highways, indicating a strong vehicular contribution, with an average concentration of 72.6 ± 39.2 ng·m^−3^. Low and medium-molecular weight PAHs were detected in serum samples at an average concentration of 43.8 ± 8.8 ng·g^−1^ of lipid (mean of ∑ individual congeners). In contrast, a high-molecular-weight PAH, benzo[a]pyrene (BaP), was detected in one participant. Pyrene (PYR) and fluoranthene (FLU) were the predominant congeners, suggesting combustion-related sources, primarily vehicular emissions. Serum PAH levels showed a correlation with the frequency of consumption of canned fish and meat, but not with short-term airborne PAH measurements. These exploratory findings suggest that dietary intake is a primary pathway of bioaccumulation during acute exposure and plays a key role in determining the parental PAHs burden during pregnancy in polluted environments. However, additional data on parent PAHs and their metabolites are needed to provide a more comprehensive assessment of cumulative exposure arising from dietary sources and chronic inhalation of airborne PAHs.

## 1. Introduction

Air quality has become a significant risk factor affecting both maternal health and fetal development, generating approximately 6.7 million premature deaths annually [1]. Atmospheric pollutants are emitted from various sources, such as vehicle exhaust, industrial activities, forest fires, and fossil fuel combustion [2]. Among them, polycyclic aromatic hydrocarbons (PAHs) are of toxicological concern due to their widespread environmental occurrence and persistence, as well as their carcinogenic and endocrine-disrupting potential [3]. Consequently, PAHs are priority environmental pollutants in urban places and in areas with significant emission sources, where vulnerable population groups, including children, the elderly, and pregnant women, may experience elevated exposure and associated health risks [4].

In humans, atmospheric PAHs are absorbed through the respiratory epithelium and rapidly enter the systemic circulation [5]. Once in the bloodstream, they can be distributed to various tissues and organs, particularly those with high lipid content, where accumulation may contribute to the body’s internal burden and potential adverse health effects [3]. Pregnant women represent a particularly vulnerable population, as physiological changes during gestation, including increased ventilation rate, altered lipid metabolism, and heightened placental perfusion, among others, may increase both internal PAH exposure and tissue distribution [6]. Therefore, understanding the internal exposure pathway is essential for linking ambient PAH levels to maternal and fetal environmental exposure.

During pregnancy, PAHs have been shown to reach the placenta and umbilical cord, resulting in direct fetal exposure [7,8]. Congeners such as naphthalene (NAP), acenaphthylene (ACY), anthracene (ANT), pyrene (PYR), benzo[b]fluoranthene (BbF), benzo[k]fluoranthene (BkF), and benzo[a]pyrene (BaP), among others, have been reported in placental tissue at concentrations ranging from 0.5 to 35 ng·mL^−1^. In addition, BaP, BbF, and dibenz[a,h]anthracene (DBA) have been associated with spontaneous preterm birth [9]. Studies in placental cell lines have shown that ANT, BbF, and BaP impair cell viability and induce necrosis [10]. Furthermore, exposure to BaP, PYR, and fluoranthene (FLU) disrupts gestational hormones, including beta-human chorionic gonadotropin (β-hCG) and progesterone, suggesting impaired endometrial receptivity and placental dysfunction [11].

Distribution of PAHs in maternal blood may facilitate accumulation in breast milk, as their lipophilic properties promote partitioning into the milk lipid fraction [12]. Consequently, infant exposure through breastfeeding may contribute to adverse health effects during early childhood development [13]. Total PAH concentrations (ΣPAH) have been quantified in breast milk at 0.71 to 378 ng·g^−1^ lipid mass in countries such as Italy [14], Portugal [15], and the Czech Republic [16]. In Colombia, breast milk PAH concentrations of up to 186.5 ng·g^−1^ lipid mass have been reported and were associated with an increased incremental lifetime cancer risk (ILCR) due to infant dietary exposure to PAHs [17].

Following inhalation exposure, PAHs can enter systemic circulation and undergo biotransformation into hydroxylated metabolites (OH-PAHs). However, the presence of unmetabolized parent compounds in blood serum reflects recent or ongoing exposure and provides critical insight into internal dose [18]. Serum PAH measurements are therefore particularly relevant for assessing exposure during pregnancy, as circulating parent compounds reflect the fraction available for maternal tissue uptake and placental transfer [19]. In addition, the presence of compounds such as pyrene (PYR) and fluoranthene (FLU) may help identify potential pollution sources, as they serve as indicators of fossil fuel combustion [20].

In a previous work conducted in Medellín, Colombia, it was reported that marked spatial differences in airborne PAH concentrations, with substantially higher levels in the southern region of the city compared to the north, coincided with a higher prevalence of adverse pregnancy outcomes, including missed abortion [21]. Although these associations do not establish causality, they underscore the potential relevance of regional air pollution to reproductive risk. Building on this evidence, the present study aims to compare the exploratory PAH concentrations in maternal blood serum across different regions of Antioquia, Colombia, providing novel data on internal exposure during pregnancy and supporting future investigations into placental bioaccumulation, lactational exposure, and maternal–fetal health outcomes.

## 2. Materials and Methods

### 2.1. Study Area

**Figure 1:**
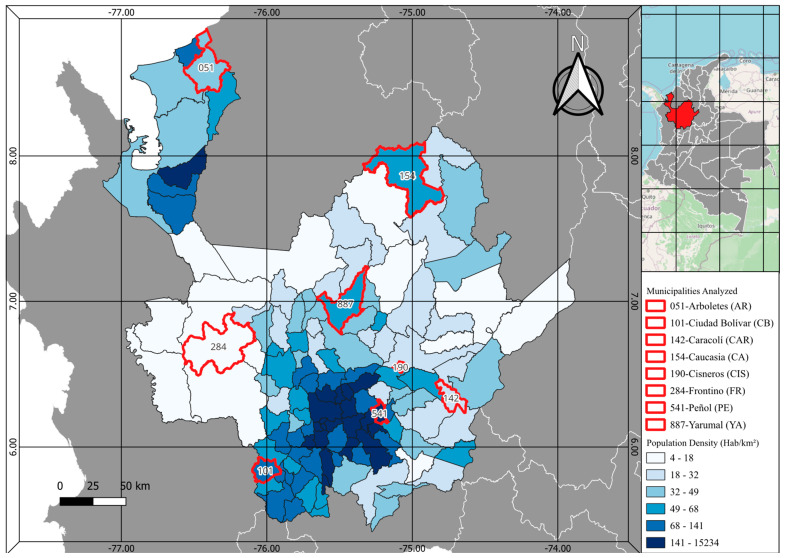
Study area. The municipalities included in the study are outlined in red. Population density (inhabitants km^−2^) across Antioquia is represented by the blue color gradient, with darker shades indicating higher population density.

Antioquia is one of the 32 departments of Colombia, covering an area of approximately 63,612 km^2^ and a population of about 7 million inhabitants. The department is geographically divided into nine regions that differ in altitude and temperature, factors that influence air pollutant dynamics and, consequently, population exposure levels. The selection of participants and environmental monitoring of PAHs were conducted in one municipality in eight regions, including Yarumal (YAR), Caucasia (CA), Cisneros (CIS), Caracolí (CAR), Arboletes (AR), Frontino (FR), Ciudad Bolívar (CB), and El Peñol (PE), during September 2025. See Figure 1.

The municipalities included in this study differ considerably in area, population size, degree of urbanization, and economic activities [22,23]. General geographical and demographic characteristics are presented in Table 1. Caucasia (CA) is the most populated municipality (93,843 inhabitants) and functions as a regional economic center, where gold mining, livestock production, agriculture, fishing, and commercial activities coexist. Yarumal (YA), one of the largest municipalities by area (753.8 km^2^), combines mining, livestock production, and commercial activities, supported by its strategic location along the main transportation corridor to northern Colombia. Frontino (FR) is characterized by mining and agricultural activities, whereas Caracolí (CAR) combines mining with livestock production despite its relatively small population (4510 inhabitants). Cisneros (CIS), the smallest municipality by area (47.9 km^2^), supports economic activities related to tourism, agriculture, livestock production, and mining. In contrast, Ciudad Bolívar (CB) is recognized for coffee production. At the same time, El Peñol (PE) and Arboletes (AR) are primarily characterized by agricultural activities and tourism, with PE focusing on avocado and tomato cultivation and AR on banana production, livestock, tourism, and local commerce. These differences in population density, economic activities, degree of urbanization, and connectivity through regional transportation networks may influence the intensity and distribution of PAH emission sources, particularly those associated with vehicular traffic, commercial activity, and fossil fuel combustion in CA, CB, AR, and PE.

### 2.2. Real-Time Monitoring of Airborne PAHs

Environmental real-time monitoring of PAHs was conducted on roads and motorways (overall vehicular traffic areas) in the selected eight municipalities during September and November 2025. Total airborne PAH concentrations were measured using a photoelectric aerosol sensor (PAS 2000; EcoChem Analytics, League City, TX, USA) equipped with a UV radiation detector. The PAS 2000 sensor uses a UV lamp to photoionized particles bound to PAHs [24]. Thus, the specific irradiation energy and wavelength of the UV lamp ensure that only particles with adsorbed PAHs emit photoelectrons, not other aerosols. The instrument’s analytical quantification range was 0–4000 ng·m^−3^, with a lower detection limit of 10 ng·m^−3^. The device was deployed in high-traffic areas located near the residential zones of participating pregnant women. It was set up to collect one data point per minute (data acquisition), and the sampling flow rate was set to 5 L·min^−1^. Real-time data were recorded using PAHDAS 3.2.0.0 software (EcoChem Analytics, League City, TX, USA) and exported as text files, which were subsequently processed and analyzed using the R Project for Statistical Computing (2025.09.0).

### 2.3. Participants and Blood Samples Collection

Conducting cohort studies in certain regions of Colombia faces significant logistical and operational challenges, including public security concerns, high rates of internal migration, and long travel distances between participants’ residences and sampling locations, which are often exacerbated by poor road conditions. These factors may contribute to participant attrition and limit recruitment and follow-up in cohort studies. Thereby, the final participants included were CA (1), CAR (3), FR (3), YAR (2), CIS (2), AR (2), PE (3), and CB (3). A total of 19 participants were included. All eligible participants received a detailed explanation of the study objectives, procedures, and potential implications, and written informed consent was obtained before inclusion. The study protocol and the survey administration were approved by the Ethics Committee N° 062024 of Corporación Universitaria Remington, ensuring compliance with ethical principles regarding risk–benefit balance and participant protection. Pregnant women were recruited during early gestation, and inclusion criteria comprised confirmed pregnancy, residence within the study area, and willingness to participate. Exclusion criteria included the presence of diagnosed chronic diseases or conditions that could interfere with PAH metabolism or exposure assessment. Participants reporting exposure to wood-burning activities, tobacco smoke, or household fuel use were excluded. Demographic and clinical information was collected for each participant, including age, body weight (BW), Body mass index (BMI), gestational age (GA), place of residence, and duration of residence in the study area (DRSA).

Maternal blood samples were collected from accredited clinical laboratories in each region following standardized clean procedures during routine prenatal clinical examinations, thereby avoiding additional invasive procedures or extra blood sampling. Blood samples were collected in two yellow-top Vacutainer^®^ tubes and centrifuged to separate serum, which was stored at 4–8 °C during transport to the analytical laboratory for PAH determination.

### 2.4. Chemicals and Reagents

A standard solution containing the 16 U.S. EPA priority PAH compounds, along with deuterated PAH internal standards, was purchased from Sigma-Aldrich (purity > 99.5%, St. Louis, MO, USA). Hexane used for analytical extraction and preparation of PAH stock solutions was obtained from Sigma-Aldrich (GC–MS SupraSolv^®^ grade, St. Louis, MO, USA). Fetal bovine serum (FBS) was also purchased from Sigma-Aldrich (purity > 99%, St. Louis, MO, USA). Silica gel 60 and sodium chloride were purchased from Merck^®^ (Darmstadt, Germany). The capillary column Rtx-5Sil MS (30 m × 0.25 mm i.d., 0.15 μm film thickness) was obtained from Restek (Pure Chromatography, Bellefonte, PA, USA).

### 2.5. Preparation of Spiked Samples and Procedural Blanks for GC/MS Analyses

Obtaining a human serum matrix free of target PAH compounds is highly challenging because low-level environmental exposure is widespread in the general population. Therefore, fetal bovine serum (FBS) was selected as a surrogate matrix for method validation, recovery experiments, and matrix-effect evaluation [25,26]. The spiked samples were prepared by transferring 1.5 mL of FBS into centrifuge tubes, then adding 50 µL of a PAH standard solution (600 ng mL^−1^), 10 mL of hexane, 500 mg of sodium chloride, and 2 mL of Milli-Q water (Type II). Blank samples were prepared in parallel using FBS without PAH standards. All centrifuge tubes were homogenized using a mechanical shaker at 200 rpm for 50 min. Samples were then centrifuged at 3000 rpm for 10 min and subsequently stored at −20 °C for 24 h to facilitate the organic phase separation by freezing-assisted extraction. The protocol flowchart and procedural details are presented in the Supplementary Materials (See Figures S1 and S2). Limits of detection (LOD) and quantification (LOQ) were estimated using the blank signal method based on ten blank samples (Equation (S1)), and recovery experiments were performed in triplicate (Supplementary Materials—See Table S3).

### 2.6. Sample Extraction and Cleanup for PAHs Analysis

The extraction procedure was adapted from a previously reported method for determining PAHs in human blood serum [27]. Aliquots of 1.5 mL of each participant’s serum were transferred into centrifuge tubes followed by the addition of 10 mL of hexane, 500 mg of sodium chloride, and 2 mL of Milli-Q water (Type I). The extraction procedure was performed according to the same protocol used for spiked samples (Section 2.5). Sample extraction was then performed according to the protocol described for the spiked samples (Section 2.5). For sample cleanup, solid-phase extraction (SPE) cartridges packed with 600 mg of silica gel 60 and 500 mg of sodium chloride were conditioned with 2 mL of hexane before use. The organic fraction obtained after extraction was passed through the SPE cartridges, then washed with an additional 2 mL of hexane to complete the cleanup. The purified organic extract was collected in an amber flask and concentrated to dryness using rotary evaporation. The dried extracts were reconstituted in hexane, transferred to chromatographic vials, and adjusted to a final volume of 500 µL. All samples and quality-control extracts were stored at −20 °C until analysis.

### 2.7. PAH Analysis by GC/MS

All extracts obtained from the organic phase were qualitatively and quantitatively analyzed using a Thermo Scientific Trace^®^ Ultra (Austin, TX, USA) gas chromatograph coupled to an ISQ mass spectrometer operated in selected ion monitoring (SIM) mode under electron impact ionization (70 eV). Separation of PAH congeners was achieved on an Rtx-5Sil MS capillary column (30 m × 0.25 mm i.d., 0.15 μm film thickness), with an initial oven temperature of 70 °C and a final temperature of 320 °C. Helium was used as the carrier gas at a constant flow rate of 2 mL·min^−1^. Calibration curves were prepared in hexane over the concentration range of 10–100 ng·mL^−1^ (Supplementary Materials—See Figure S3). Quantification was performed using deuterated internal standards (ISTDs), as previously described [28]. More details for mass spectrum analysis are presented in the Supplementary Materials (See Table S2).

### 2.8. Data Analysis

Data visualization and statistical analyses were performed using GraphPad Prism 7.0 and the R Project for Statistical Computing. Real-time PAH data were additionally processed using Microsoft Excel (2016, v16.0) and R. Chromatographic data acquired from the Thermo Scientific Trace^®^ Ultra GC–MS system were processed using Xcalibur 1.6 software. The Shapiro–Wilk test (n < 50) was performed to assess the normality of PAH congeners and socio-demographic characteristics (*p* > 0.05). The Shapiro–Wilk test revealed non-normal distributions for some variables, and thus, non-parametric tests were applied throughout. The Wilcoxon Signed-Rank test was used to analyze the differences between paired congeners. Additionally, the Wilcoxon Rank-Sum test was employed to assess differences between independent congeners. Correlation matrices among variables were constructed using Pearson correlation analysis on municipality-level means. The significance criterion was set at *p* < 0.05 for all statistics.

## 3. Results

### 3.1. Characteristics of the Participants

The study included volunteer pregnant women aged 18 to 40 years. Detailed participant characteristics are provided in Table 2 and Table S3.

### 3.2. GC/MS Parameters and Quality Control

Separation on the Rtx-5Sil MS column provided adequate chromatographic resolution, enabling reliable identification and quantification of individual PAH congeners and their corresponding deuterated internal standards (ISTDs). Representative chromatograms are provided in the Supplementary Materials (see Figure S4). Recovery values for individual congeners ranged from 60 to 120%, except for acenaphthene (ACE). Overall, these recovery ranges were acceptable for most analytes according to established standard methods [29]. In addition, the low relative standard deviations (RSDs) indicated good method precision. Further details are presented in Table 3. The linear ranges and coefficients of determination (*r*^2^) reported in Table 3 demonstrated suitable performance for quantitative analysis. Linearity plots are provided in the Supplementary Materials (see Figure S3).

### 3.3. Total Airborne PAHs Analysis

**Figure 2:**
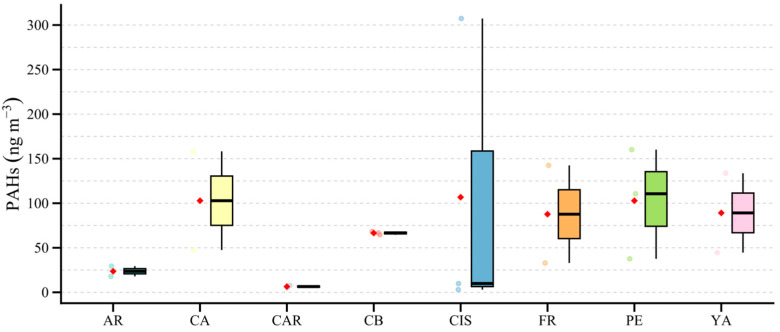
Box-and-whisker plots of airborne PAH concentrations across the municipalities included in the study. Boxes represent the interquartile range, the central line indicates the median, red diamonds represent means, and circles indicate outliers.

Higher levels of total PAHs were found in CB, CA, PE, and FR, which have higher traffic contributions. Differences between airborne PAHs from regions are presented in Figure 2.

### 3.4. Levels of PAH Congeners in Blood Serum from Pregnant Women

**Figure 3:**
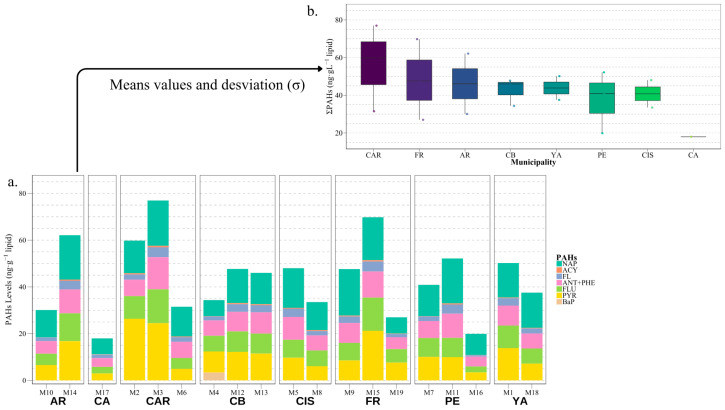
(**a**) Blood serum concentrations of individual PAH congeners measured in single blood samples across the study municipalities. (**b**) Box-and-whisker plots showing the distribution of total serum PAH concentrations by municipality; boxes represent the interquartile range, the central line indicates the median, and whiskers denote data dispersion.

Detection frequencies confirmed the predominance of low- and medium-molecular-weight PAH congeners in maternal serum samples. Naphthalene (NAP), acenaphthylene (ACY), fluorene (FL), anthracene + phenanthrene (ANT + PHE), fluoranthene (FLU), and pyrene (PYR) were detected in 19 of 19 samples (100%), whereas benzo[a]pyrene (BaP) was detected in only one participant (5.3%). Levels are reported in ng·g^−1^ of lipid (more details in Supplementary Materials—Table S4). See Figure 3.

Correlation analysis showed a negative association between airborne PAH concentrations and serum PAH levels in pregnant women (*r* = −0.71), suggesting that short-term ambient measurements may not fully capture individual exposure patterns (Figure S5). Similarly, r < 0.50 was obtained in the correlation analysis between serum PAH concentrations and socio-demographic variables, such as time of residence in the study area. These results may be further influenced by factors such as mobility patterns, indoor exposures, and metabolic variability, highlighting the need for future studies incorporating PAH metabolites. However, serum PAH concentrations showed a positive correlation with certain dietary variables, such as the frequency of meat and canned fish consumption (r = 0.78 and 0.83, respectively).

Shapiro–Wilk test indicated normal distribution for most PAH congeners and socio-demographic variables (*p*-value > 0.05), except for key variables, such as *p*yrene (*p*-value = 0.027), gestational age (*p*-value = 0.006), and duration of residence (*p*-value = 0.040), showing significant deviations from normality (*p*-value < 0.05). See Table 2. Wilcoxon rank-sum tests confirmed that all targeted PAH congeners were significantly detected above the baseline (*p* < 0.001), except for BaP. Furthermore, paired Wilcoxon signed-rank tests showed distinct internal exposure profiles among congeners. Significant differences in concentration were observed across all evaluated pairs, including NAP vs. PYR (*p*-value = 0.0313), FL vs. FLU (*p*-value < 0.001), and ANT+PHE vs. BaP (*p*-value < 0.001), all of which retained their statistical significance after Benjamini–Hochberg (BH) false discovery rate adjustment (*p*-value < 0.05). See Supplementary Materials—Table S5.

**Figure 4:**
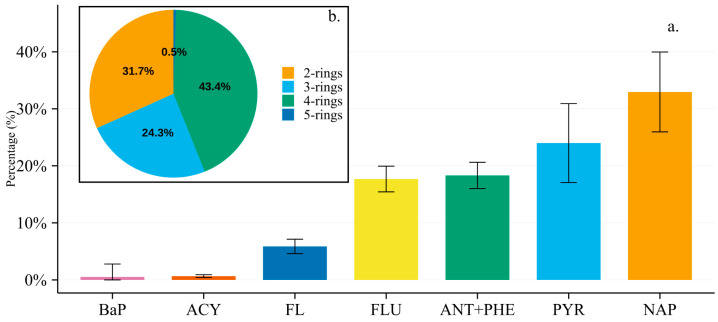
(**a**) Percentage contribution of individual PAH congeners detected in blood serum samples. (**b**) Percentage distribution of PAHs according to the number of aromatic rings.

Consistent with these findings, low and medium-molecular-weight PAH congeners were the predominant contributors to the total PAH burden in maternal blood serum, suggesting similar exposure profiles. These compounds are commonly associated with petrogenic sources [17], whereas high-molecular-weight PAHs are typically linked to pyrogenic processes [30]. The distribution of PAHs by number of aromatic rings is shown in Figure 4.

## 4. Discussion

### 4.1. Analytical Methods

An analytical method using liquid–liquid extraction with water and sodium chloride was employed to isolate the target PAHs. This methodology was adapted from previously published protocols and further optimized to enhance sample cleanup and recovery efficiency [27,31]. Recovery values for spiked samples ranged from 60% to 120%, consistent with previously reported ranges (88.0–113.8%), with relative standard deviations (*RSDs*) below 15%, indicating acceptable precision. The method exhibited approximately a fourfold increase in sensitivity compared with the referenced approaches. Consequently, the analytical protocol proved suitable for PAH determination in blood serum, achieving a linear dynamic range of 10–100 ng·mL^−1^ and good analytical accuracy (*RSD* < 15%). Given the heterogeneous exposure levels among populations residing in air-polluted urban environments, certified FBS was used as a procedural blank to minimize matrix effects and background interference. This approach contributed to a substantial improvement in the LOQ.

### 4.2. Blood Levels of PAHs and Human Implications

This study observed positive associations between dietary variables and serum PAH concentrations, suggesting a possible contribution of trophic transfer to the internal PAH burden. However, the present study focused exclusively on parent PAH compounds, which are generally considered indicators of recent exposure. Previous studies have reported no clear associations between the frequency of meat and fish consumption and blood PAH levels, likely because PAHs are rapidly metabolized into epoxy and hydroxylated derivatives and subsequently excreted through urine [19].

Levels of PAHs in blood serum were observed at 43.8 ± 8.8 ng·g^−1^ of lipid, which is almost thirty times lower compared with a previous report in Shanghai, China (1290 ng g^−1^ lipid), where the traffic contribution is higher [19]. The PAH concentrations observed in this study were substantially lower than those reported for perinatal outcomes, such as anencephaly, spina bifida, encephalocele and neural tube defects (5400 to 6300 ng·g^−1^ of lipid in blood serum) [32].

Some municipalities, particularly CA, CB, and FR, exhibited higher airborne PAH concentrations, reflecting the influence of local anthropogenic activities. However, no significant correlation was observed between airborne PAH concentrations and serum PAH levels. These municipalities are characterized by intensified human activities, including mining operations, agricultural production, commercial activities, and higher traffic density [23], all of which are recognized sources of low- and medium-molecular-weight PAHs derived from fossil fuel combustion and other combustion-related processes [20]. Therefore, further analysis should be conducted to estimate inhalation exposure.

In contrast, municipalities such as AR, where lower serum PAH concentrations were detected, also exhibited comparatively lower airborne PAH levels and are predominantly characterized by agricultural activities, which are generally associated with lower PAH emissions than combustion-dominated sources. Although causality cannot be established from this exploratory study, the consistency observed between environmental and biological measurements supports the plausibility that air pollution contributes to PAH exposure during pregnancy. These findings also highlight the importance of region-specific emission sources in shaping internal PAH burdens. Furthermore, the transfer of PAHs among environmental compartments, including water, food products, and the trophic chain, warrants further investigation. While no significant correlations were observed between airborne and serum PAH concentrations, positive associations were identified between serum PAH levels and certain dietary variables, suggesting that non-inhalation exposure pathways may also contribute to the observed internal burden. Finally, the predominance of low- and medium-molecular-weight PAH congeners in serum samples is consistent with exposure to petrogenic inputs and fossil fuel-related emissions.

Municipalities intersected by major national highways and characterized by intense vehicular activity may therefore experience greater PAH exposure and body burden among residents. Consistent with this interpretation, our previous work identified an increased risk of inhalation exposure among populations residing in traffic-impacted residential areas [21]. Similarly, higher PAH exposure levels have been reported in more industrialized settings [33]. The predominance of FLU and PYR in serum samples further supports combustion-related sources, particularly vehicular emissions, as major contributors to exposure. Indeed, PAHs such as PHE, FLU, and PYR have been associated with traffic-related particles and road environments, where volatile PAHs emitted from motor vehicles could be adsorbed onto particulate matter [20].

The detection of PAHs in maternal blood serum reflects their absorption and systemic distribution, corresponding to the initial phases of the absorption–distribution–metabolism–excretion (ADME) process [3]. Therefore, the blood levels of PAHs may reflect an early stage in their transfer to other biological matrices, such as the placenta and breast milk, which pose a future risk to neonates [9,34]. In Colombia, PAH concentrations in breast milk averaging approximately 186.6 ng·g^−1^ lipid have been reported [17], which are higher than the average serum PAH levels of 43.8 ± 8.8 ng·g^−1^ of lipid observed in the present study (mean of ∑ individual congeners). This discrepancy is likely attributable to the strong affinity of PAHs for the lipid fraction of breast milk.

### 4.3. Air Pollution and Possible Implications for Human Bioconcentration

In general, high-molecular-weight and carcinogenic PAHs are more frequently detected in human blood following exposure to intensive industrial activities, tobacco smoke, or prolonged domestic combustion processes, such as wood burning [27]. Accordingly, the detection of BaP in a single pregnant participant may be associated with passive smoking exposure, as the participant reported frequent household exposure to tobacco smoke. This interpretation is supported by previous studies showing that smokers and coke-oven workers may exhibit up to 65% of high-molecular-weight PAHs relative to total blood PAHs [27].

As shown in Figure 4, NAP showed the highest proportion of detected congeners in blood serum; additionally, 99.4% of all identified PAHs consisted of low- and medium-molecular-weight congeners containing two to four aromatic rings. This compositional profile is characteristic of mixtures influenced by petrogenic sources, which are naturally enriched in 2–3 ring PAHs and their alkylated homologs due to the geologic maturation of organic matter into petroleum. In contrast, pyrogenic sources typically generate higher proportions of high-molecular-weight PAHs (>4 rings) through thermal cracking and aromatization, with heavy congeners accounting for up to 58% of total PAHs in occupationally exposed populations such as coke-oven workers and smokers [27]. The near-absence of >4-ring PAHs in our cohort, except for the single detection of BaP, indicates that maternal exposure in these municipalities is dominated by low-temperature, petrogenic-influenced fossil-fuel emissions and moderate combustion, rather than intense high-temperature industrial combustion or heavy tobacco smoke [35]. The isolated detection of BaP in one participant with frequent household tobacco smoke exposure aligns with the known pyrogenic origin of high-molecular-weight PAHs in tobacco smoke and coke-oven environments.

**Figure 5:**
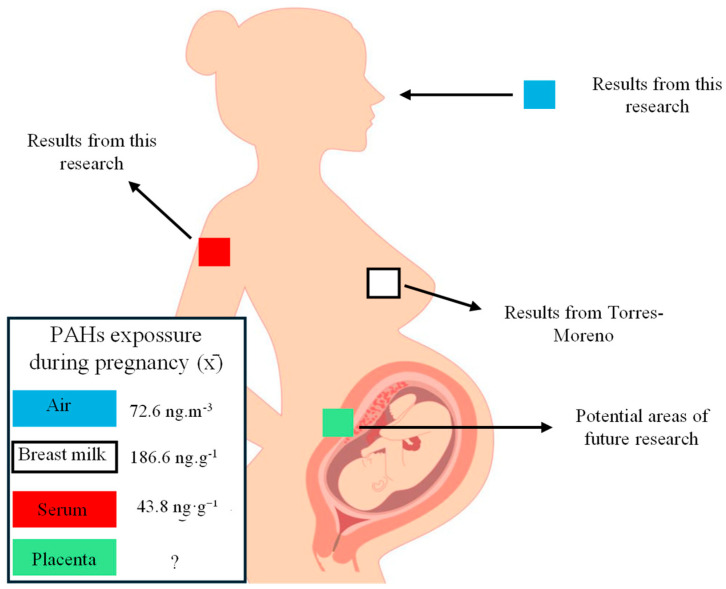
Graphical representation of PAHs exposure during pregnancy in Colombia, summarizing reported PAH levels in different biological matrices, including ambient air, maternal blood serum (this study), and breast milk [17].

The average airborne PAH concentration measured in the present study was approximately 72.64 ng·m^−3^, supporting inhalation as a relevant exposure pathway. During pregnancy, increased ventilatory volume may further enhance respiratory uptake and internal accumulation of PAHs [35]. Nevertheless, additional exposure routes, including ingestion of contaminated drinking water, consumption of charred foods, and dietary intake of fish, may also contribute to PAHs bioconcentration. A summary of available data on PAHs exposure during pregnancy and lactation in Colombia is presented in Figure 5.

As illustrated in Figure 5, future risk assessment efforts should prioritize placental analysis, as fetal bioconcentration may result from maternal exposure to PAHs that circulate in the blood. In the present study, PAHs detected in maternal serum were predominantly low- and medium-molecular-weight compounds, which are not classified as carcinogenic by the U.S. Environmental Protection Agency (EPA) (see Figure 4) [36]. Nevertheless, certain PAHs, such as NAP and PYR, have been associated with adverse reproductive outcomes and may act as endocrine-disrupting chemicals. These compounds have been reported to reduce estradiol (E2) and anti-Müllerian hormone (AMH) levels and increase follicle-stimulating hormone (FSH) concentrations in umbilical cord serum [37].

Prenatal exposure to PAHs has been associated with miscarriage, low birth weight, intrauterine growth restriction (IUGR), and spontaneous abortion [38,39,40,41]. For example, a case–control study conducted in China reported that elevated maternal blood PAH concentrations were associated with a fourfold increase in the risk of missed abortion during early pregnancy [42]. In this context, the present study contributes to the growing body of evidence highlighting the importance of improved exposure assessment and preventive strategies to address adverse obstetric and perinatal outcomes associated with airborne PAH exposure. Consistent with these observations, our previous in vitro studies demonstrated that PAHs disrupt gestational hormones, including β-hCG and progesterone, potentially impairing endometrial receptivity and placental function, thereby increasing the risk of embryonic loss and IUGR [21].

The findings of the correlation analyses suggest that duration of residence may serve as an exploratory covariate associated with serum PAH levels during pregnancy. Although the observed associations should be interpreted with caution given the exploratory sample size, this correlation cannot distinguish whether elevated serum PAHs reflect acute exposure events during the sampling period, repeated episodic exposures among residents of high-exposure municipalities, or truly chronic exposure patterns. This uncertainty highlights a key limitation of air quality monitoring combined with parent PAH measurements only. To properly assess whether residential history predicts chronic cumulative burden, future studies must integrate extended environmental monitoring, detailed residential/occupational histories, PAH metabolite measurements, and larger patient cohorts. Such an approach would improve the characterization of exposure sources and better capture the complexity of maternal–environment interactions in polluted settings. Overall, PAHs detected in maternal blood serum represent the absorption and initial systemic distribution of recent/ongoing inhalation exposure during pregnancy. Following absorption, these parent compounds undergo rapid biotransformation and excretion. Still, before elimination, they distribute to other biological matrices, including tissues, placenta, and breast milk, thereby creating a window of exposure risk for maternal-fetal health [19]. The presence of PAHs in maternal serum during pregnancy indicates the available fraction for maternal tissue uptake and transplacental transfer, with potential adverse implications for fetal development and early neonatal health.

### 4.4. Limitations of This Research

In Colombia, internal migration driven by security concerns, economic opportunities, and social factors may hinder the assessment of chronic environmental exposure, limiting the reconstruction of long-term individual exposure histories. Consequently, the serum PAH concentrations reported in this study primarily reflect recent or ongoing exposure. To partially address this limitation, duration of residence was incorporated as an exposure-related variable, allowing a more refined interpretation of residential exposure. In this context, airborne PAH measurements provided additional information regarding potential exposure sources.

This study should be considered exploratory due to the limited sample size and the complexity of assessing individual PAH exposure during pregnancy in certain regions in Colombia, ultimately reducing sample size and statistical power.

### 4.5. Future Research

Future investigations should also evaluate indoor exposure sources, including biomass combustion for cooking, tobacco smoke, and household fuel use, as these are recognized contributors to PAH exposure and may significantly influence PAH burdens. Also, studies on the bioconcentration of PAHs in the trophic chain and their implications for human cumulative exposure through feeding should be conducted.

## 5. Conclusions

This study demonstrates the presence of PAHs in maternal blood serum during early pregnancy across different regions of Antioquia, Colombia, highlighting ongoing internal exposure to airborne pollutants. Low- and medium-molecular-weight PAHs were consistently detected, with PYR and FLU as the predominant congeners, indicating combustion-related sources, particularly vehicular emissions, as the main contributors. High-molecular-weight PAHs were largely absent, with BaP detected in only one participant, likely linked to specific exposure scenarios such as passive smoking.

Although serum PAH levels showed spatial patterns broadly consistent with airborne PAH distributions, no positive association was observed with short-term ambient measurements. In contrast, serum PAH concentrations were positively correlated with dietary factors such as canned fish and meat, suggesting that feeding exposure may be relevant to determining internal PAH burden during pregnancy. While this significant correlation does not imply causality, our results suggest important bioconcentration of substances primarily emitted into the atmosphere that may eventually accumulate in the trophic chain. However, this is an exploratory study, and further experiments should be conducted, including trophic chain analysis.

Finally, the most frequently detected PAH congeners are not classified as carcinogenic; however, growing evidence indicates that several low- and medium-molecular-weight PAHs may exert endocrine-disrupting effects and contribute to adverse obstetric and perinatal outcomes. The presence of PAHs in maternal serum represents the fraction available for systemic distribution to other biological matrices, with potential implications for fetal and neonatal exposure. Overall, maternal serum emerges as a valuable biomonitoring matrix for assessing prenatal PAH exposure in environmentally impacted regions. Future studies should prioritize longitudinal exposure assessment, metabolite characterization, and maternal–fetal biomonitoring approaches to improve exposure study and support public health strategies aimed at protecting maternal and fetal health.

## Figures and Tables

**Table 1:** Geographic and demographic characteristics of the study municipalities in Antioquia, Colombia.

Variable		AR	CAR	CA	CIS	CB	PE	FR	YA
Mean temperature (°C)		27.7	23.7	28	24	17.1	18.4	20.6	21.9
Mean precipitation (mm)		184.2	232	217.4	167.5	283.4	246.3	716.9	291.8
Mean pressure (hPa)		998.5	934.8	1002.5	846	840.8	814.9	889.5	815.4
Area (km^2^)		775.2	267.7	1465.5	47.9	264.6	120.5	1410.2	753.8
Population	Total	31,707	4510	93,843	10,092	27,573	23,827	21,778	41,884
	Urban (%)	38.5	61.4	89.3	87.6	61.7	54.6	44.3	71.8
	Rural (%)	61.5	38.6	10.7	12.4	38.3	45.4	55.7	28.2
	Male (%)	49.7	50.4	48.2	47.7	50.3	48.6	50.3	48.9
	Female (%)	50.3	49.6	51.8	52.3	49.7	51.4	49.7	51.1

**Table 2:** Socio-demographic characteristics of the population studied.

Variable	Category	AR	CA	CAR	CB	CIS	FR	PE	YA	Total
Age range (years)	15–20	1 (5.2%)	0 (0%)	1 (5.2%)	0 (0%)	0 (0%)	0 (0%)	0 (0%)	1 (5.2%)	3 (15.8%)
	21–25	1 (5.2%)	0 (0%)	0 (0%)	2 (10.5%)	1 (5.2%)	3 (15.7%)	1 (5.2%)	1 (5.2%)	9 (47.4%)
	26–30	0 (0%)	0 (0%)	1 (5.2%)	1 (5.2%)	1 (5.2%)	0 (0%)	2 (10.5%)	0 (0%)	5 (26.3%)
	31–35	0 (0%)	0 (0%)	0 (0%)	0 (0%)	0 (0%)	0 (0%)	0 (0%)	0 (0%)	0 (0%)
	36–40	0 (0%)	1 (5.2%)	1 (5.2%)	0 (0%)	0 (0%)	0 (0%)	0 (0%)	0 (0%)	2 (10.5%)
Gestational trimester	1st Trimester (1–12 weeks)	0 (0%)	0 (0%)	0 (0%)	0 (0%)	0 (0%)	0 (0%)	0 (0%)	0 (0%)	0 (0%)
	2nd Trimester (13–27 weeks)	1 (6.2%)	1 (6.2%)	3 (18.7%)	2 (12.5%)	2 (12.5%)	3 (18.7%)	1 (6.2%)	2 (12.5%)	15 (93.8%)
	3rd Trimester (28–40 weeks)	0 (0%)	0 (0%)	0 (0%)	0 (0%)	0 (0%)	0 (0%)	1 (6.2%)	0 (0%)	1 (6.2%)
Ethnicity	None	0 (0%)	1 (5.2%)	3 (15.7%)	3 (15.7%)	2 (10.5%)	3 (15.7%)	3 (15.7%)	2 (10.5%)	17 (89.5%)
	Indigenous	1 (5.2%)	0 (0%)	0 (0%)	0 (0%)	0 (0%)	0 (0%)	0 (0%)	0 (0%)	1 (5.3%)
	Afro-descendant	1 (5.2%)	0 (0%)	0 (0%)	0 (0%)	0 (0%)	0 (0%)	0 (0%)	0 (0%)	1 (5.3%)
Educational attainment	Primary	0 (0%)	1 (5.2%)	1 (5.2%)	0 (0%)	0 (0%)	0 (0%)	1 (5.2%)	0 (0%)	3 (15.8%)
	Secondary	1 (5.2%)	0 (0%)	1 (5.2%)	3 (15.7%)	1 (5.2%)	2 (10.5%)	1 (5.2%)	1 (5.2%)	10 (52.6%)
	Technical	0 (0%)	0 (0%)	1 (5.2%)	0 (0%)	1 (5.2%)	1 (5.2%)	0 (0%)	1 (5.2%)	4 (21.1%)
	University	1 (5.2%)	0 (0%)	0 (0%)	0 (0%)	0 (0%)	0 (0%)	1 (5.2%)	0 (0%)	2 (10.5%)
Place of residence	Urban (municipal seat)	1 (5.2%)	1 (5.2%)	0 (0%)	1 (5.2%)	1 (5.2%)	1 (5.2%)	3 (15.7%)	1 (5.2%)	9 (47.4%)
	Semi-urban (village)	1 (5.2%)	0 (0%)	3 (15.7%)	2 (10.5%)	1 (5.2%)	2 (10.5%)	0 (0%)	1 (5.2%)	10 (52.6%)
BMI category (pre-pregnancy)	Normal weight (18.5–24.9)	0 (0%)	0 (0%)	2 (10.5%)	0 (0%)	1 (5.2%)	0 (0%)	0 (0%)	0 (0%)	3 (15.7%)
	Overweight (25.0–29.9)	2 (10.5%)	1 (5.2%)	1 (5.2%)	2 (10.5%)	0 (0%)	3 (15.7%)	3 (15.7%)	2 (10.5%)	14 (73.6%)
	Obese (30.0–34.9)	0 (0%)	0 (0%)	0 (0%)	1 (5.2%)	1 (5.2%)	0 (0%)	0 (0%)	0 (0%)	2 (10.5%)
BMI category (gestational)	Normal weight (18.5–24.9)	0 (0%)	0 (0%)	2 (11.1%)	0 (0%)	0 (0%)	0 (0%)	0 (0%)	0 (0%)	2 (11.1%)
	Overweight (25.0–29.9)	2 (11.1%)	1 (5.5%)	1 (5.5%)	2 (11.1%)	0 (0%)	3 (16.6%)	3 (16.6%)	2 (11.1%)	14 (77.7%)
	Obese (30.0–34.9)	0 (0%)	0 (0%)	0 (0%)	1 (5.5%)	1 (5.5%)	0 (0%)	0 (0%)	0 (0%)	2 (11.1%)
BW (kg)	<60 kg	1 (5.5%)	0 (0%)	1 (5.5%)	0 (0%)	0 (0%)	0 (0%)	0 (0%)	0 (0%)	2 (11.1%)
	60–69.9 kg	0 (0%)	1 (5.5%)	2 (11.1%)	1 (5.5%)	0 (0%)	2 (11.1%)	1 (5.5%)	1 (5.5%)	8 (44.4%)
	70–79.9 kg	1 (5.5%)	0 (0%)	0 (0%)	0 (0%)	0 (0%)	1 (5.5%)	2 (11.1%)	1 (5.5%)	5 (27.7%)
	>80 kg	0 (0%)	0 (0%)	0 (0%)	2 (11.1%)	1 (5.5%)	0 (0%)	0 (0%)	0 (0%)	3 (16.6%)
DRSA (years)	<5 years	1 (7.1%)	1 (7.1%)	1 (7.1%)	0 (0%)	1 (7.1%)	1 (7.1%)	1 (7.1%)	0 (0%)	6 (42.8%)
	5–10 years	0 (0%)	0 (0%)	0 (0%)	0 (0%)	1 (7.1%)	0 (0%)	0 (0%)	0 (0%)	1 (7.1%)
	11–20 years	0 (0%)	0 (0%)	0 (0%)	0 (0%)	0 (0%)	0 (0%)	0 (0%)	1 (7.1%)	1 (7.1%)
	>20 years	1 (7.1%)	0 (0%)	1 (7.1%)	2 (14.2%)	0 (0%)	0 (0%)	2 (14.2%)	0 (0%)	6 (42.8%)

Body weight (BW) and Body mass index (BMI), and Duration of residence in the study area (DRSA).

**Table 3:** Chromatographic, analytical, and validation parameters for the determination of PAH congeners by GC–MS.

PAH Congeners	Rt (min)	Quantification ion (Q1)	Recovery (%)	RSD	LOD (ng·mL^−1^)	LOQ (ng·mL^−1^)	Range (ng·mL^−1^)	*r* ^2^
* Naphthalene—D8	4.34	136						
Naphthalene (NAP)	4.80	128	115.5	9.8	3.17	9.52	10–100	0.9973
Acenaphthylene (ACP)	6.69	152	120.2	4.4	2.51	7.53	10–100	0.9993
* Acenaphthene-D10	6.89	162						
Fluorene (FL)	7.60	166	68.7	1.7	2.51	7.52	10–100	0.9952
* Phenanthrene-D10	9.42	188						
Phenanthrene (PHE)	8.47	178	79.1	0.9	2.99	8.98	10–100	0.9989
Anthracene (ANT)	8.47	178	79.1	0.9	2.99	8.98	10–100	0.9989
Fluoranthene (FLU)	12.50	202	79.7	2.3	3.00	9.00	10–100	0.9941
Pyrene (PYR)	14.70	202	117.9	12.5	3.29	9.87	10–100	0.9969
Benz[a]anthracene (BaA)	18.63	228	98.2	10.4	1.07	3.212	10–100	0.9950
* Chrysene-D12	19.51	240						
Chrysene (CHRY)	19.74	228	74.8	2.7	1.36	4.09	10–100	0.9932
Benzo[b,] fluoranthene (BbF)	22.85	252	101.5	5.9	0.472	1.415	10–100	0.9972
Benzo[k]fluoranthene (BkF)	20.85	252	101.5	5.9	0.472	1.415	10–100	0.9972
Benzo[a]pyrene (BaP)	22.72	252	86.9	0.4	2.90	8.71	10–100	0.9986
* Perylene-D12	23.00	264						
Indeno[1,2,3-cd]pyrene (IND)	26.01	276	64.5	4.3	1.77	5.32	10–100	0.9992
Dibenz[a,h]anthracene (DahA)	26.60	278	60.5	7.6	2.13	6.40	10–100	0.9995
Benzo[ghi]perylene (BghiP)	27.66	276	80.5	2.7	2.63	7.89	10–100	0.9900

Retention time (Rt), target ions, recovery, relative standard deviation (RSD), limits of detection (LOD), limits of quantification (LOQ), linear range, and coefficients of determination (r^2^). The deuterated congeners (internal standards) are presented with an asterisk at the top. The Ace did not show the acceptable recovery. Thus, this congener was not included for analysis.

## Data Availability

The original contributions presented in this study are included in the article and Supplementary Materials. Further inquiries can be directed to the corresponding author.

## Supplementary Materials

The following supporting information can be downloaded at: https://www.mdpi.com/article/10.3390/jox16040124/s1, Figure S1: Flowchart. Figure S2: Analytical details. Figure S3: Linearity and equation for quantification purposes. Figure S4: Chromatogram for PAHs analysis. Figure S5: The matrix of correlation. Figure S6: (a) Socio-demographic characteristics of the population studied. (b) Dietary habits of included participants. Equation (S1): LOD and LOQ. Table S1: Data for LOD and LOQ analysis. Table S2: Chromatography details for PAHs analysis. Table S3: Descriptive statistics: Measures of central tendency, dispersion, and relative standard deviation (RSD). Table S4: Variables to adjustment for lipid content. Table S5: Wilcoxon Signed and Sum Rank test results.
