# Supplementary material for: Pregnancy Exposure to Polycyclic Aromatic Hydrocarbons (PAHs): Exploratory Comparative Levels in Blood Serum Samples from Different Regions in Antioquia, Colombia
Source: J Xenobiot. 2026 Jul 2;16(4):124. doi: 10.3390/jox16040124 (PMC13398110; doi:10.3390/jox16040124)
Supplement: Supplementary file 1 [file jox-16-00124-s001.zip › jox-4314766-supplementary.pdf]

Supplementary Materials: Pregnancy Exposure to Polycyclic Aromatic Hydrocarbons (PAHs): Exploratory Comparative Levels in Blood Serum Samples from Different Regions in Antioquia, Colombia

Jhon Fredy Narváez-Valderrama [1,*], Juan José García-Londoño [2], Juan David González-Calderón [3], Yileni Argoti-Ospina [1], Gabriel Jaime Maya [4], Jorge L. Gallego [5], Ana Luisa Urrego [3] and Carlos Daniel Ramos-Contreras [6]

[1] Grupo de Investigación Ingeniar, Facultad de Ingenierías, Corporación Universitaria Remington, Calle 51 No. 51-27, Medellín, Colombia; yileni.argoti.1439@miremington.edu.co
[2] Grupo de Investigación Materialografía, Transición Energética y Ambiente (MATREA), Facultad de Ingenierías, Corporación Universitaria Remington, Calle 51 No. 51-27, Medellín 050012, Colombia;
juan.garcia.3750@miremington.edu.co
[3] Grupo de Investigaciones Biomédicas UniRemington, Corporación Universitaria Remington, Calle 51 No. 51-27, Medellín 050012, Colombia; juan.gonzalez01@uniremington.edu.co (J.D.G.C.); anaurrego10@gmail.com (A.L.U.)
[4] Grupo de Investigaciones y Mediciones Ambientales GEMA, Facultad de Ingenierías, Universidad de Medellín, Carrera 87 No. 30-65, Medellín 050026, Colombia; gjmaya@udemedellin.edu.co
[5] Biodiversity, Biotechnology and Bioengineering Research Group—GRINBIO, Department of Engineering, University of Medellin, Medellín 050026, Colombia; jlgallego@udemedellin.edu.co
[6] Grupo de Investigación en Gestión y Modelación Ambiental—GAIA, Facultad de Ingeniería - Escuela de Microbiologia, Universidad de Antioquia—U.de.A, Calle 70 No 52-21, Medellín 050010, Colombia; daniel.ramos@udea.edu.co
* Correspondence: jhon.narvaez@uniremington.edu.co

Introduction

Exposure to airborne polycyclic aromatic hydrocarbons (PAHs) during pregnancy has been associated with adverse obstetric and perinatal outcomes, including miscarriage, low birth weight, intrauterine growth restriction, and spontaneous abortion. This study includes the analysis of maternal blood samples for PAHs detection. The supplementary material (SM), the data validation, quantification, identification, statistical treatment and specific analytical steps are presented.

Flowchart for analytical methods

The methods included biological samples treatment, liquid–liquid extraction, phases separation, clean up, volume reduction (concentration), quality control and GC/MS analysis

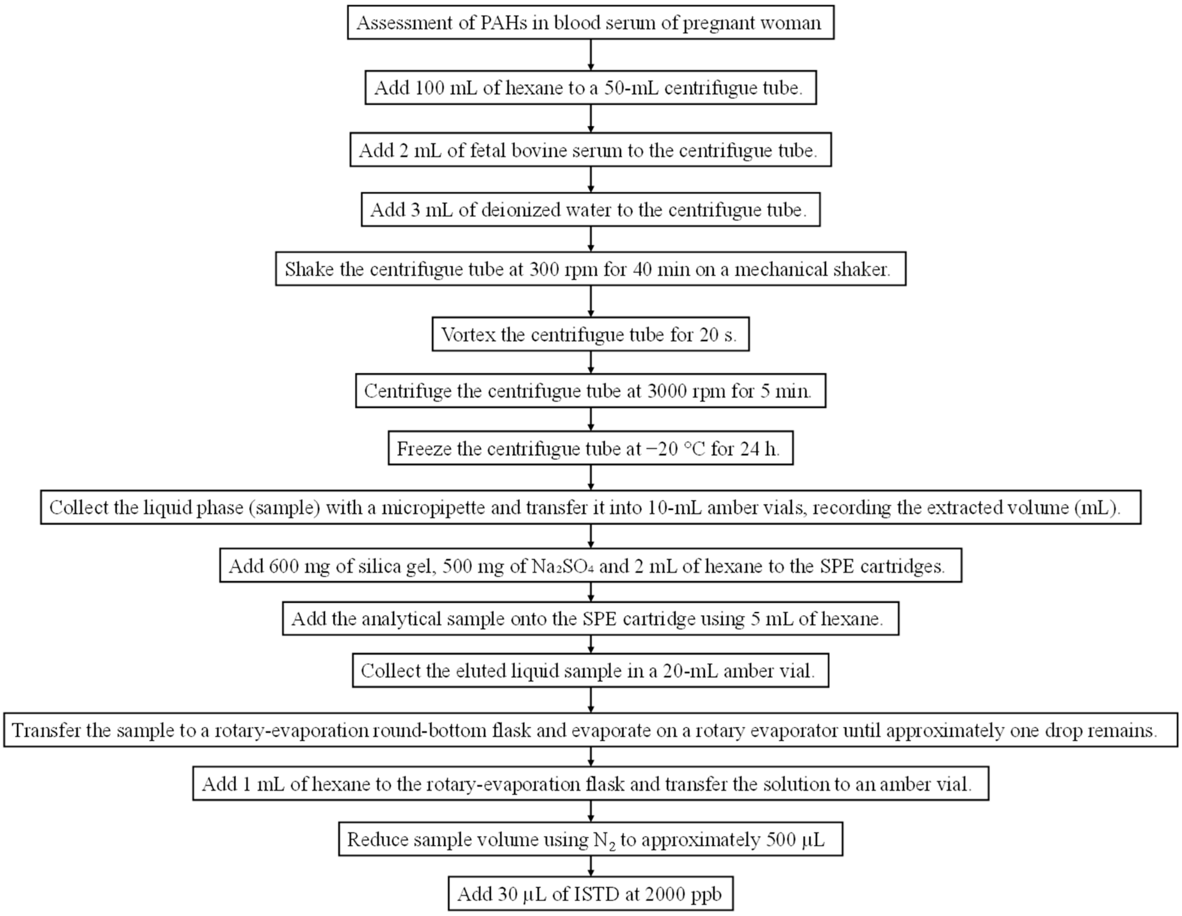

Figure S1. Flowchart.

Step by step protocol and photos

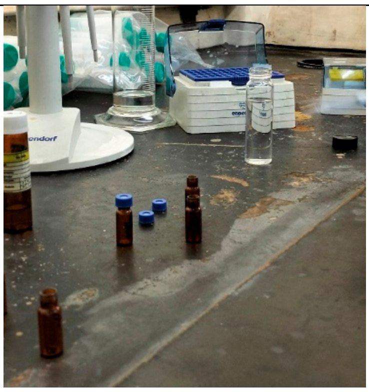	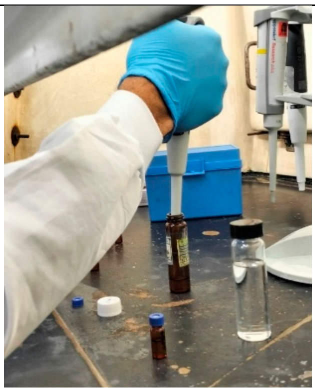
Calibration curves	Standard solution (Stock) 1000 $ng.mL^{-1}$ and 400 $ng.mL^{-1}$

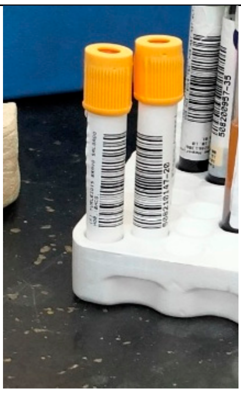	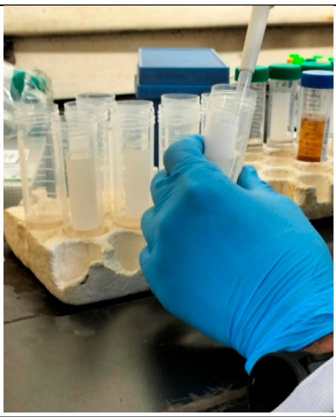
Blood serum samples	LOD and LOQ simples – Fetal bovine serum (FBS)
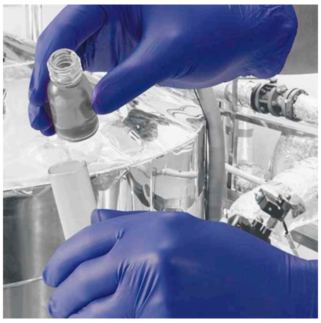	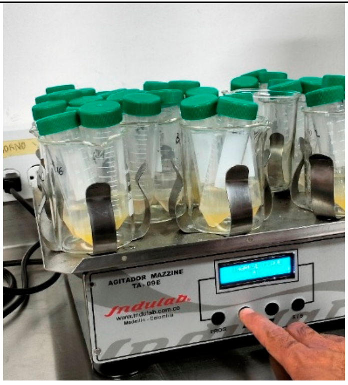
Preparing SPE cartridge using silica gel 60 and Na_2SO_4	Shaking samples
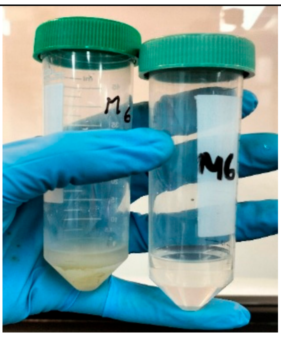	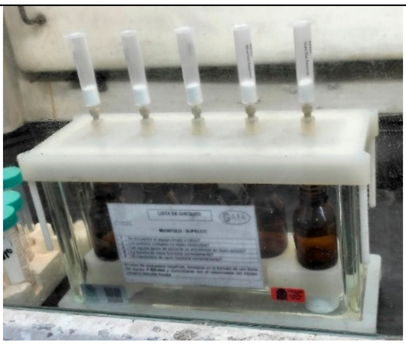
Liquid/liquid extraction	Clean up by SPE using a manifold
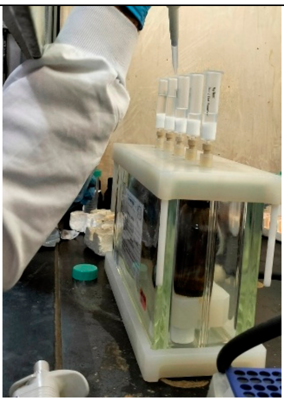	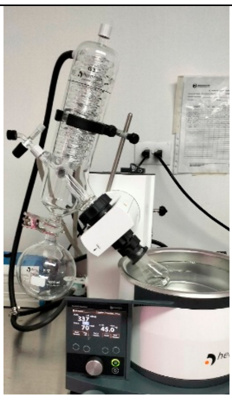
Clean up	Reducing extraction volume by rotary evaporator

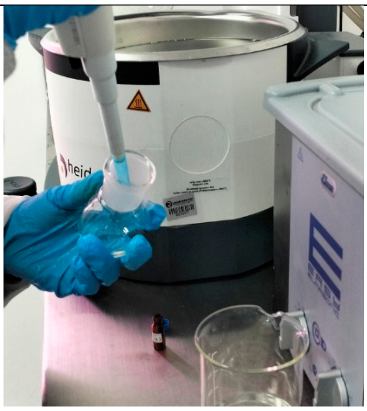	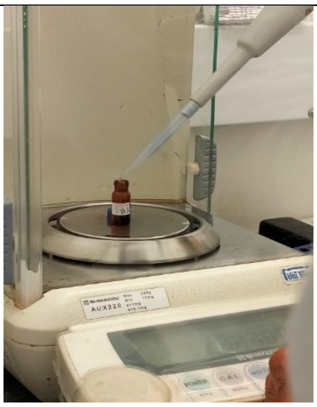
Samples recovery from rotary flask	Analytical control by gravimetry method
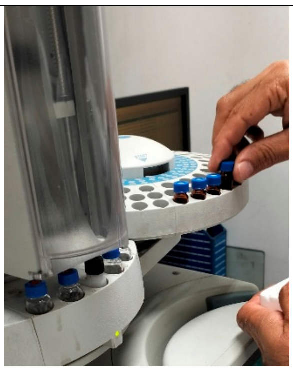	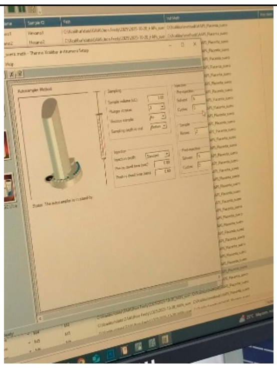
GC-MS analysis	GC analytical method

Figure S2. Analytical details.

LOD and LOQ by noise signal method

The sensitivity of the methods was tested by blanks analysis. Areas were estimated in the range of ±1 second around Rt. Thereby, limit of detection (LOD) and the limit of quantification were determined by following equations:

Equation S1. LOD and LOQ.

$$LOD = \frac{3.3\sigma}{S} \qquad LOQ = \frac{10\sigma}{S}$$

where σ: Standard deviation and S: Slope of each calibration curve

Table S1. Data for LOD and LOQ analysis.

Congeners	NAP	ACY	ACE	FL	ANT +PHE	FEN	FLU	PYR	BaA	CRY	BkF+ BbF	BaP	IND	DahA	BghiP
Replicates	0.601	0.030	1.810	0.168	0.610	0.610	0.200	0.410	0.010	0.030	0.008	0.018	0.009	0.009	0.0214
	0.600	0.039	1.830	0.170	0.623	0.623	0.210	0.410	0.012	0.032	0.005	0.028	0.005	0.005	0.0220
	0.590	0.041	1.801	0.174	0.583	0.583	0.196	0.389	0.015	0.029	0.004	0.026	0.004	0.004	0.0220
	0.581	0.020	1.820	0.159	0.598	0.598	0.198	0.410	0.017	0.031	0.007	0.029	0.010	0.010	0.0019
	0.578	0.021	1.825	0.156	0.600	0.600	0.213	0.415	0.014	0.035	0.009	0.026	0.009	0.009	0.0210
	0.580	0.039	1.811	0.172	0.587	0.587	0.220	0.390	0.016	0.041	0.007	0.024	0.007	0.007	0.0210
	0.590	0.029	1.800	0.170	0.578	0.578	0.210	0.400	0.014	0.032	0.010	0.040	0.013	0.013	0.0220
	0.580	0.041	1.810	0.174	0.618	0.618	0.209	0.388	0.013	0.023	0.002	0.034	0.019	0.019	0.0210
SD	0.009	0.009	0.011	0.007	0.016	0.016	0.008	0.011	0.002	0.005	0.003	0.007	0.005	0.005	0.0069
Slope	0.0096	0.0117	0.0111	0.0089	0.0181	0.0181	0.0091	0.0112	0.0071	0.0124	0.0187	0.0074	0.0089	0.0074	0.0087
LOD	3.172	2.511	3.213	2.508	2.993	2.993	3.001	3.289	1.071	1.364	0.472	2.903	1.773	2.132	2.632
LOQ	9.516	7.532	9.640	7.523	8.979	8.979	9.003	9.866	3.212	4.092	1.415	8.710	5.318	6.396	7.895

The standard deviation is presented.

Calibration curves for PAH congeners (ISTD method)

The Kolmogorov–Smirnov test was applied for assessment the normality test which indicated that the variables fitted into Gaussian distribution (p-value= 0.5135 > 0.05). By contrast, one-way ANOVA showed that the mean relative area for PAH congeners was not equal in the levels of the calibration curves (p-value= $3.09 \times 10^{-6} < 0.05$). Levene's test supported the assumption of homoscedasticity across the levels of the calibration curve (p-value= 0.478 > 0.05). Thereby, data indicate that the residual variance remained constant. Finally, the Variance Inflation Factor (VIF) was evaluated for all PAH congeners which showed a high degree of collinearity among between variables. See calibration curves

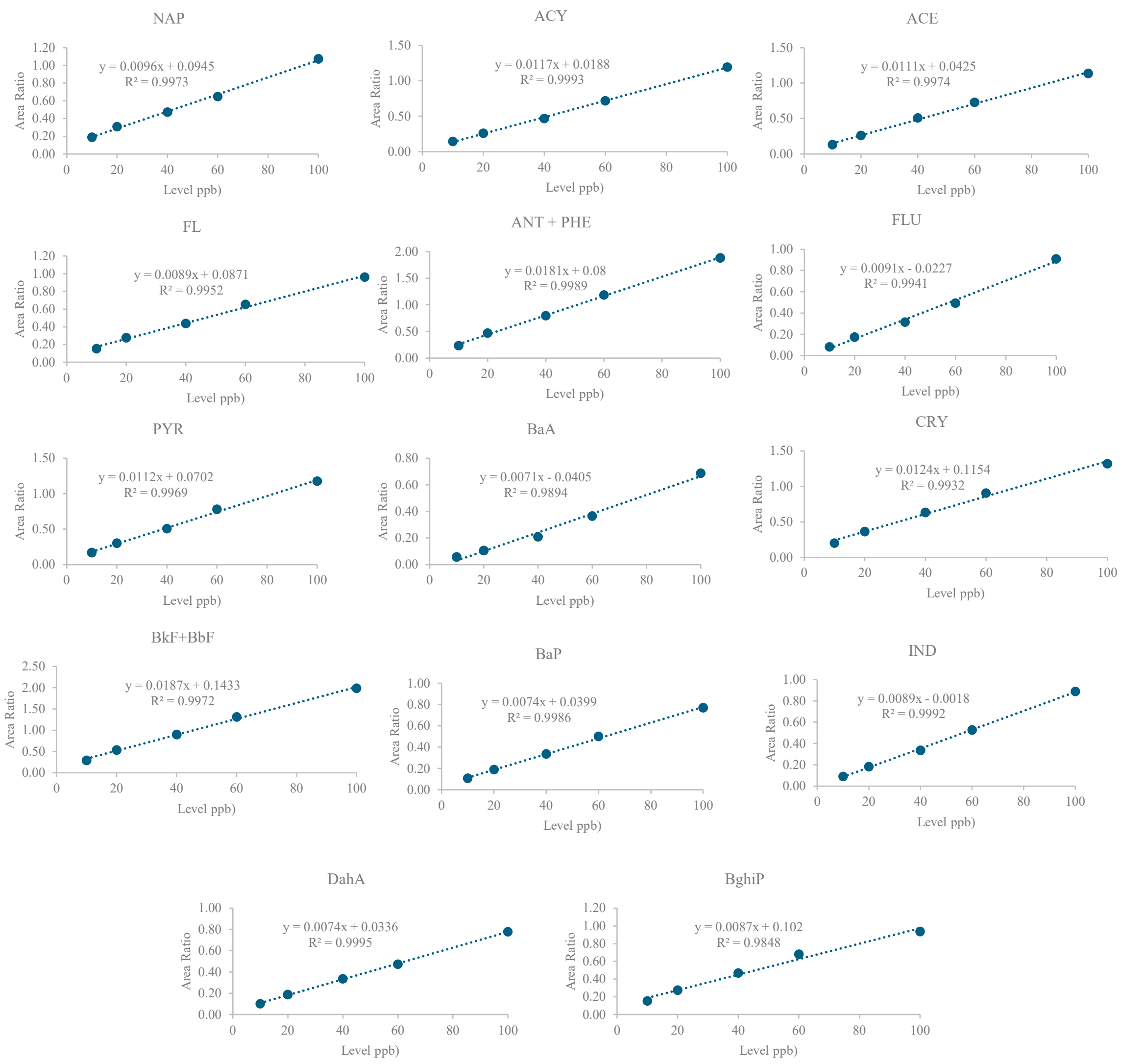

Figure S3. Linearity and equation for quantification purposes.

S5. GC/MS Method retention times and quantification ions

Table S2. Chromatography details for PAHs analysis.

	PAH Congeners	tR (min)	Q1	Q2	Q3
2	*Naphthalene – D8	4.34	136	137	108
3	Naphthalene (NAP)	4.80	128	102	129
5	Acenaphthylene (ACP)	6.69	152	153	76
6	Acenaphthene (ACE)	6.80	162	164	80
7	*Acenaphthene-D10	6.89	153	152	76
8	Fluorene (FL)	7.60	166	165	139
9	*Phenanthrene-D10	9.42	188	189	160
10	Phenanthrene (PHE)	8.47	178	176	76
11	Anthracene (ANT)	8.47	178	179	176
12	Fluoranthene (FLU)	12.50	202	203	101
13	Pyrene (PYR)	14.70	202	203	101
14	Benz[a]anthracene (BaA)	18.63	228	226	114
15	*Chrysene-D12	19.51	240	236	120
16	Chrysene (CHRY)	19.74	228	226	114

17	Benzo[b,] fluoranthene (BbF)	22.85	252	253	126
18	Benzo[k]fluoranthene (BkF)	20.85	252	253	126
20	Benzo[a]pyrene (BaP)	22.72	252	253	126
21	*Perylene-D12	23.00	264	260	132
23	Indeno[1,2,3-cd] pyrene (IND)	26.01	278	276	139
24	Dibenz[a,h]anthracene (DahA)	26.60	276	278	138
25	Benzo[ghi]perylene (BghiP)	27.66	276	274	138

tR: Retention time, Q1: Quantification ion, Q2: Quality ion 1, Q3: Quality ion 2.

Selected chromatographs

The chromatographic method allowed an adequate resolution for congeners separation. Analysis of PAH congeners and deuterated compounds used as ISTD were carried out in a single method.

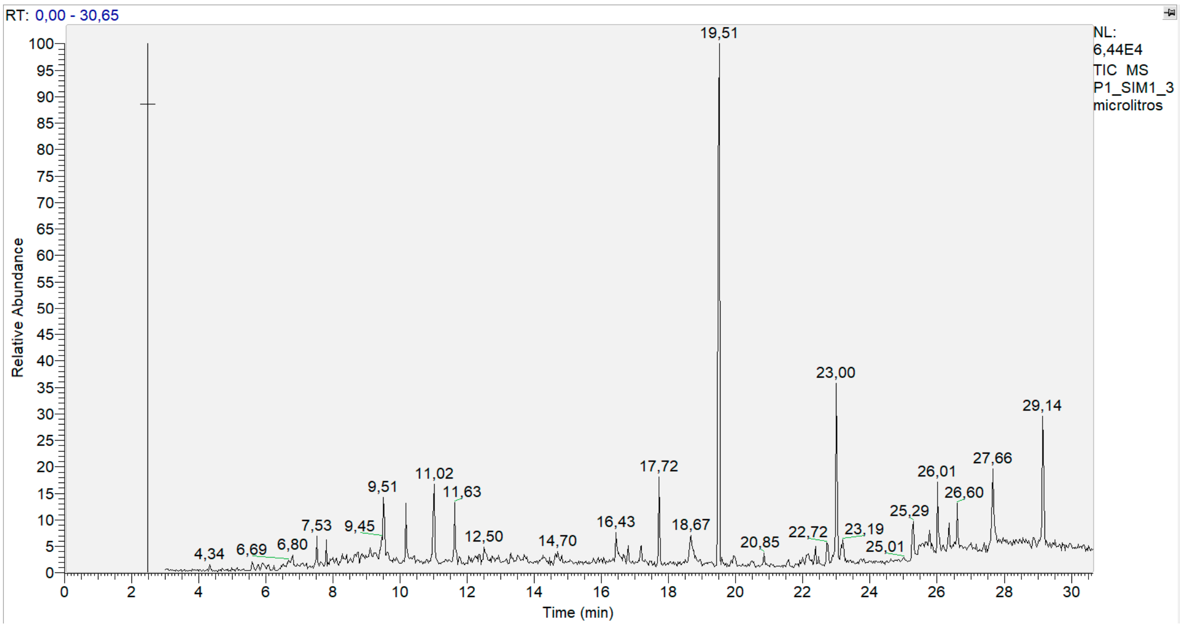

Figure S4. Chromatogram for PAHs analysis.

Analytes and ISTDs are presented.

Matrix of correlation

Positive correlation is fitted to blue color while the negative one is fitted to red. The matrix of correlation was tested by Pearson correlation coefficients.

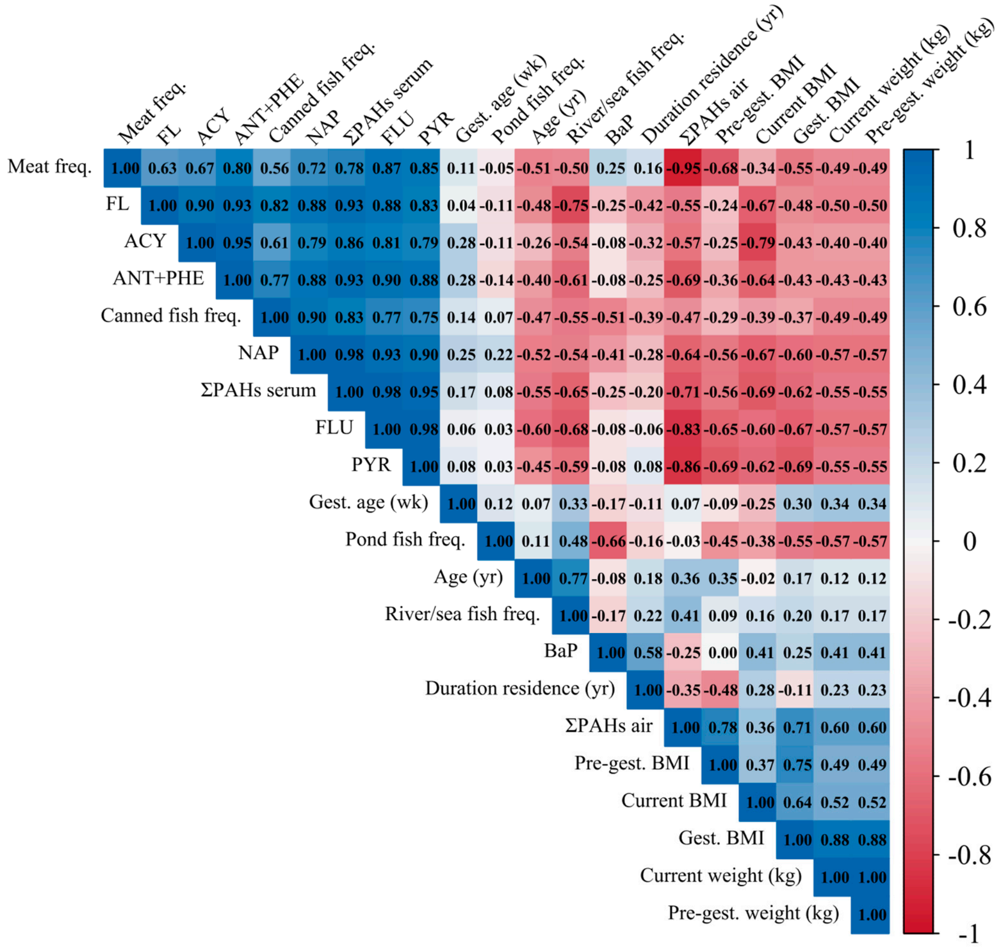

Figure S5. The matrix of correlation.

Descriptive statistics.

Table S3. Descriptive statistics: Measures of central tendency, dispersion, and relative standard deviation (RSD).

Variables	n	Min	Q1	Median	Mean	Q3	Max	SD	CV (%)	Skewness	Kurtosis	Missing	Weight (S-W)	*p-value* (S-W)
NAP	19	6.856	11.838	13.988	13.923	17.698	19.934	4.317	31.01	-0.255	2.047	0	0.9254	0.1427
ACY	19	0.056	0.151	0.336	0.307	0.452	0.607	0.168	54.7	-0.03	1.795	0	0.947	0.3506
FL	19	0.599	1.8	2.283	2.59	3.407	4.33	1.075	41.5	0.077	1.954	0	0.9568	0.5109
ANT+PHE	19	3.731	6.427	7.318	7.827	9.435	13.738	2.529	32.31	0.399	2.845	0	0.9756	0.8809
FLU	19	2.551	6.147	7.702	7.87	9.21	14.5	3.253	41.33	0.418	2.877	0	0.9539	0.4592
PYR	19	2.983	6.868	9.694	11.176	13.012	26.357	6.697	59.92	0.953	3.121	0	0.8854	0.0267
BaP	19	0	0	0	0.179	0	3.399	0.78	435.89	3.695	17.056	0	NA	NA
Age (years)	19	16	21.5	24	25.263	28.5	39	6.154	24.36	0.879	3.448	0	0.907	0.0651
Gestational age (weeks)	19	12	13.5	15	17.474	21.5	31	5.651	32.34	0.919	2.805	0	0.8488	0.0064
Current BMI	19	18.8	25.2	27.8	27.084	29.45	33.4	3.42	12.63	-0.485	3.262	0	0.9696	0.7688
Current weight (kg)	18	54	62.875	68.2	70.711	78.7	87	10.037	14.19	0.173	1.899	1	0.9533	0.4784
Pre-gestational weight (kg)	18	50	62	63.5	66.361	74	86	9.846	14.84	0.44	2.398	1	0.9302	0.196

Height (m)	18	1.5	1.538	1.62	1.6	1.64	1.71	0.064	4.01	-0.239	1.918	1	NA	NA
Gestational BMI	18	22.2	25.45	28.05	27.544	29.475	33.4	2.85	10.35	0.017	2.559	1	0.9799	0.9495
Pre-gestational BMI	18	20.5	23.7	25.5	25.878	28.875	30.9	3.22	12.44	-0.027	1.869	1	0.9456	0.3607
DRSA (years)	14	1	4	13	14.595	22.75	39	12.903	88.41	0.348	1.782	5	0.8688	0.0403

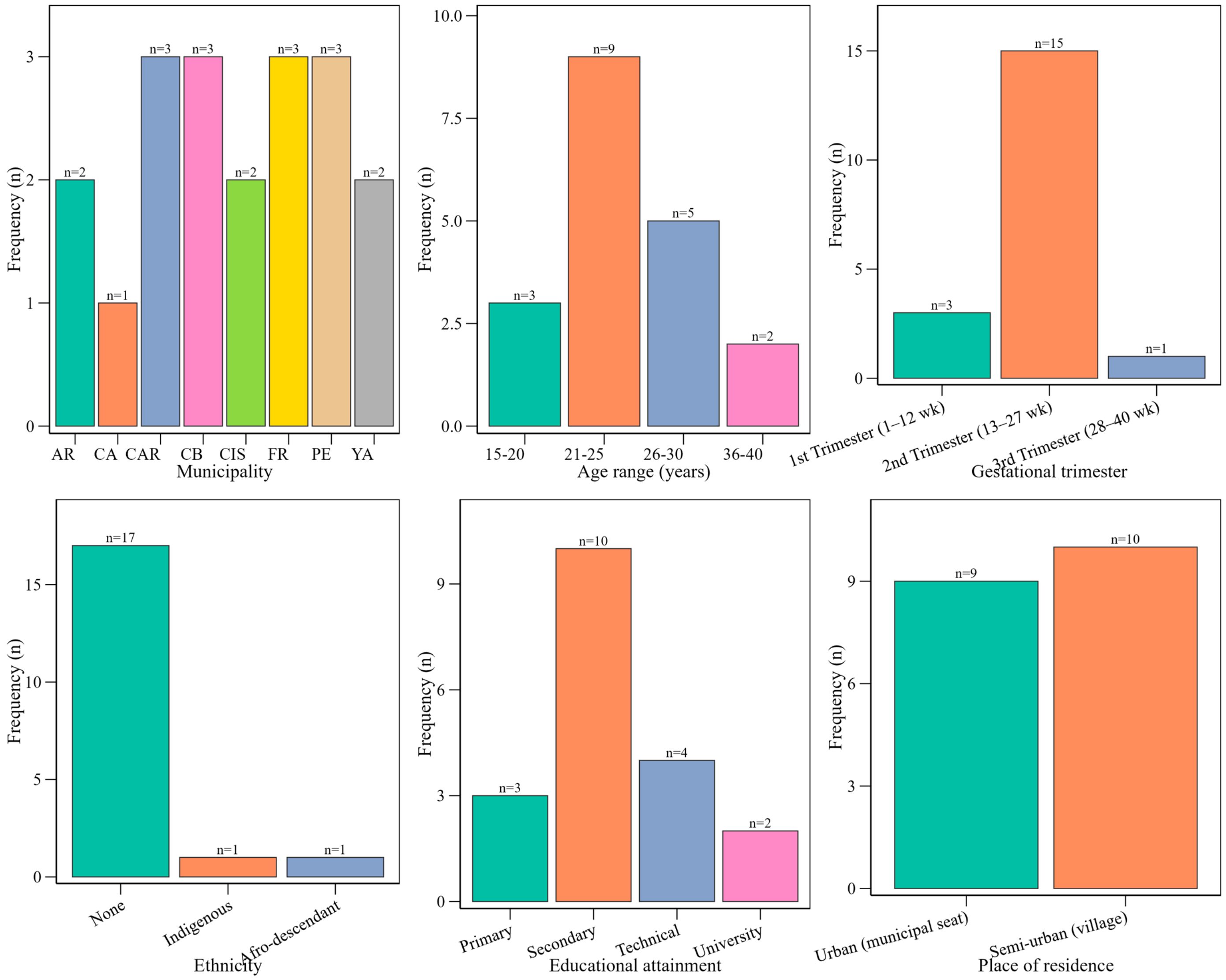

Figure S6. a. Socio-demographic characteristics of the population studied.

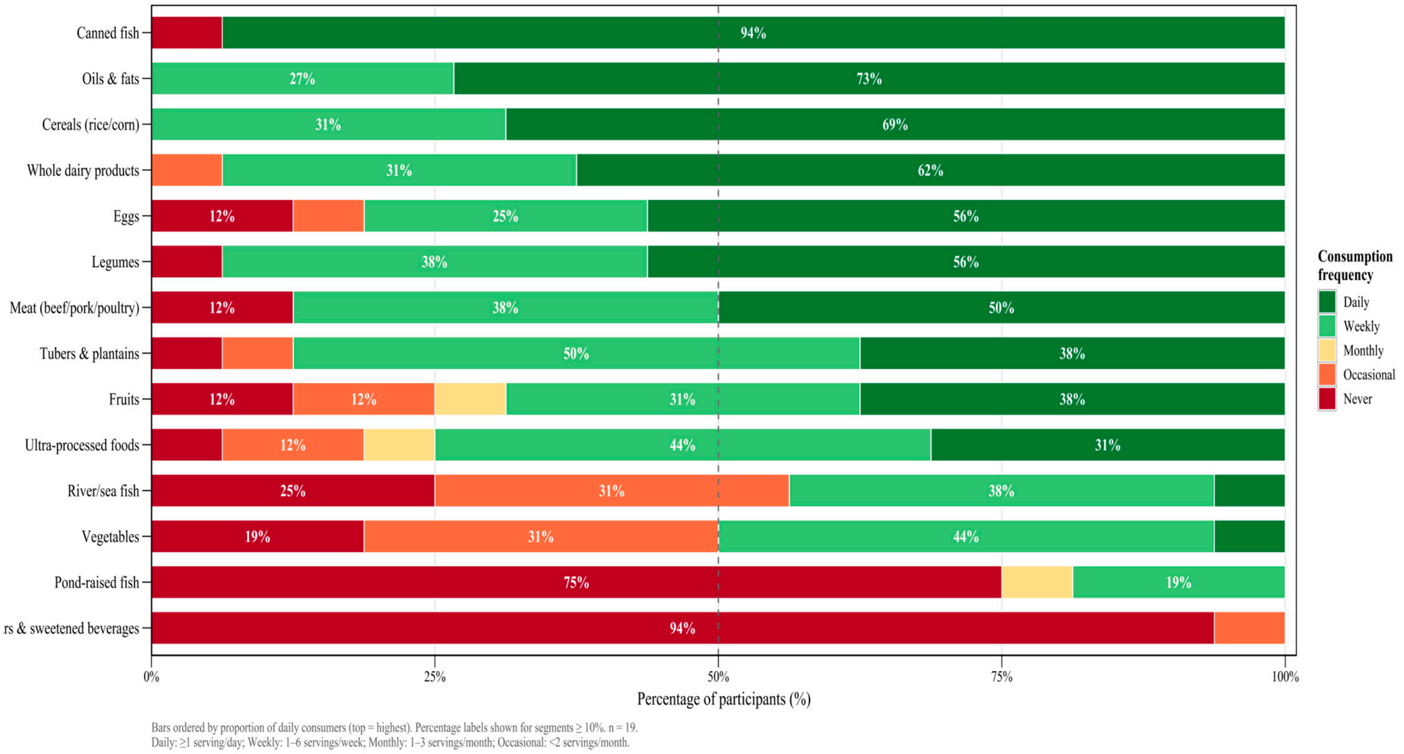

Figure S6. b. Dietary habits of included participants.

Determination of PAHs in blood serum as ng. g^{-1} of lipid.

Table S4. Variables to adjustment for lipid content.

ID	Municipality	Total cholesterol	Triglycerides	Total lipids (mL)	PAHs (ng. g^{-1} for lipid)
M1	YA	181.00	189.00	4.08	50.19
M10	AR	213.00	180.00	4.29	30.10
M11	PE	172.00	150.00	3.52	52.21
M12	CB	214.00	164.00	4.11	47.73
M13	CB	208.00	138.00	3.74	46.03
M14	AR	184.00	136.00	3.47	62.13
M15	FR	167.00	104.00	2.92	69.81
M16	PE	236.00	149.00	4.15	19.92
M17	CA	309.00	238.00	5.95	17.99
M18	YA	246.00	176.00	4.57	37.58
M19	FR	289.00	238.00	5.75	27.00
M2	CAR	248.00	218.00	5.10	59.82
M3	CAR	150.00	70.10	2.34	77.00
M4	CB	226.00	199.00	4.65	34.38
M5	CIS	204.00	106.00	3.31	48.05
M6	CAR	198.00	193.00	4.30	31.54
M7	PE	170.00	117.00	3.10	40.91
M8	CIS	195.00	180.00	4.11	33.53
M9	FR	164.00	106.00	2.91	47.64

Non-parametric test.

Table S5. Wilcoxon Signed and Sum Rank test results.

Analytical treatment	n	V	*p-value*
Rank-Sum			
NAP	19	190	1.00E-04
ACY	19	190	1.00E-04
FL	19	190	1.00E-04
ANT+PHE	19	190	1.00E-04
FLU	19	190	1.00E-04
PYR	19	190	1.00E-04
BaP	19	1	0.5
Signed-Rank test			
NAP vs. PYR	19	149	0.0313
FL vs. FLU	19	0	1.00E-04
ANT+PHE vs. BaP	19	190	1.00E-04
